# Tacrolimus-Related Cerebral Microbleeds after Lung Transplantation

**DOI:** 10.1155/2013/708961

**Published:** 2013-12-02

**Authors:** L. Mechtouff, F. Piegay, J. Traclet, F. Philit, P. Boissonnat, M. Hermier, I. Durieu, T.-H. Cho, N. Nighoghossian, J.-F. Mornex

**Affiliations:** ^1^Hospices Civils de Lyon, Hôpital Pierre Wertheimer, 59 Boulevard Pinel, 69677 Bron, France; ^2^Université de Lyon, 69003 Lyon, France; ^3^Université Lyon 1, 69003 Lyon, France; ^4^Hospices Civils de Lyon, Hôpital Louis Pradel, 69677 Bron, France; ^5^CNRS UMR 5220, INSERM U1044, CREATIS, 69622 Villeurbanne, France; ^6^INSA de Lyon, 69622 Villeurbanne, France; ^7^Hospices Civils de Lyon, Centre Hospitalier Lyon-Sud, 69495 Pierre Bénite, France; ^8^INRA UMR 754, Rétrovirus et Pathologie Comparée, 69007 Lyon, France

## Abstract

Posterior reversible encephalopathy syndrome is a well-known complication of treatment by tacrolimus. We report 2 cases of lung transplant recipients treated with tacrolimus who developed cerebral microbleeds on T2∗-weighted sequences in the acute setting of posterior reversible encephalopathy syndrome. Cerebral microbleeds may be a marker of tacrolimus-induced vasculopathy that may be detected earlier by neuropsychological and magnetic resonance imaging monitoring in transplant recipients treated with tacrolimus.

## 1. Introduction

Posterior reversible encephalopathy syndrome (PRES), although rare (1.6%/year), is a well-recognized and severe cerebral complication of the treatment by tacrolimus [1]. Tacrolimus could exert a direct toxicity on the endothelial cells leading to the alteration of the blood-brain barrier and the release of potent vasoconstrictor resulting in vasospasm and hypoperfusion [2].

Cerebral microbleeds (CMBs) are unusual in the setting of PRES and have seldom been reported in tacrolimus-treated patients. We recently observed CMBs on MRI in two-lung-transplant recipients treated with tacrolimus in the acute setting of PRES.

## 2. Case 1

**Figure 1:**
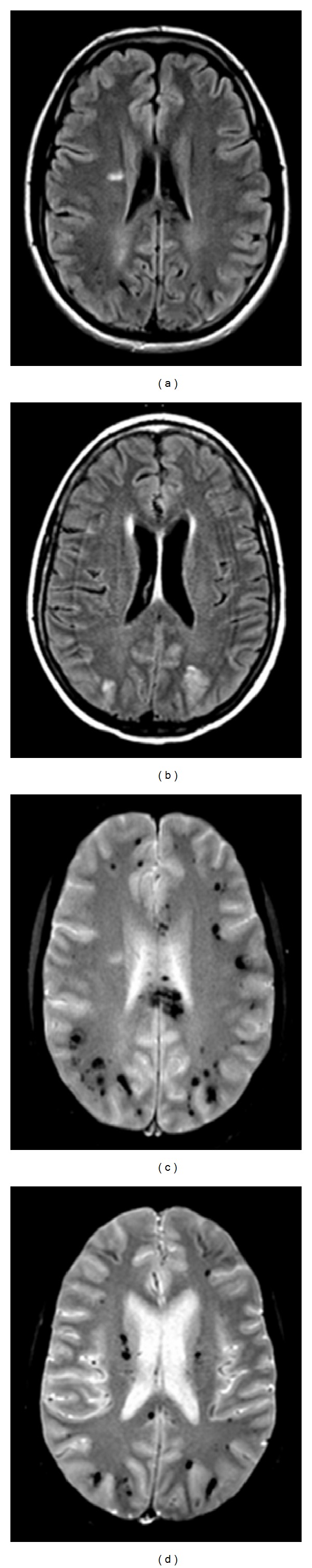
Axial FLAIR images of the 2 patients (a, b, resp.) showing right periventricular hyperintensity in case 1 and bilateral occipital hyperintensities in case 2; T2*-weighted sequences of the 2 patients (c, d, resp.) showing both cerebral microbleeds in the cortico-subcortical junction of the two cerebral hemispheres associated with cerebral microbleeds in corpus callosum in case 1 and frontal subarachnoid hemorrhage in case 2.

A 19-year-old cystic fibrosis patient with a history of diabetes underwent double lung transplantation. He was started on tacrolimus (10 mg/d), prednisone, and mycophenolate mofetil. Four months later, he developed a visual seizure secondary generalised. On admission, blood pressure was 160/105 mmHg. The tacrolimus blood trough level (CO) was 8.8 *μ*g/L, total cholesterol was 3.25 mmol/L, and creatinine was 71 *μ*mol/L. A computed tomography (CT) scan showed right parietal and bilateral occipital hypodensities without acute hemorrhage. Magnetic resonance imaging (MRI) showed right parietal and bilateral occipital hyperintensities on fluid-attenuated-inversion-recovery (FLAIR) images with high apparent diffusion coefficient (ADC) and multiple CMBs on T2*-weighted sequences in the cortico-subcortical junction of the two cerebral hemispheres and corpus callosum (Figure 1) without arterial abnormality. Cerebral spinal fluid (CSF) and electroencephalogram (EEG) were normal. The dosage of tacrolimus was decreased and everolimus was initiated. The patient did not experience further seizures. Control MRI performed one month later showed complete regression of hypersignal on FLAIR images but the persistence of CMBs on T2*-weighted sequences.

## 3. Case 2

A 26-year-old cystic fibrosis patient underwent double lung transplantation with a troubled course and a long stay in the intensive care unit and a secondary neuropathy. Immunosuppression consisted of tacrolimus (16 mg/d) and prednisolone. She was admitted 3 months later for a generalized seizure and prolonged altered consciousness. On admission, her mean blood pressure was 45 mmHg, cardiac rate was 135/minute, and temperature was 38.5°C. Glasgow scale was 9/15. Laboratory evaluation revealed a white blood cells (WBC) count of 750/mm^3^, C-reactive protein (CRP) of 133 mg/L, procalcitonin of 45 *μ*g/L, creatinine clearance of 36 mL/min, tacrolimus level (CO) of 4.1 *μ*g/L, magnesium of 0.64 mmol/L, and total cholesterol of 4.81 mmol/L. CT scan was normal. MRI showed bilateral parietooccipital, cerebellous, and thalamic hyperintensities on FLAIR images without restriction of ADC, multiple CMBs in the cortico-subcortical junction of the two cerebral hemispheres, internal capsule and cerebellum, and frontal subarachnoid hemorrhage on T2*-weighted sequences (Figure 1). *Klebsiella pneumoniae* was found in blood and urine analysis. Transesophageal echocardiography and CSF analysis were normal. An EEG showed generalized slowing and bilateral triphasic slow waves predominantly located in the anterior regions. She was treated with antibiotics. The dosage of tacrolimus was decreased and everolimus was initiated. She regained alertness 2 hours after her admission and had no more seizures but she described visual disturbances which lasted for 24 hours. She was apyretic within 48 hours; CRP and WBC count were normal 6 days later. The follow-up MRI 2 months later revealed complete regression of FLAIR lesions but the persistence of CMBs and subarachnoidal hemorrhage on T2*-weighted sequences.

## 4. Discussion

We report two cases of multiple CMBs, an unusual MRI feature, in lung transplant recipients treated with tacrolimus at “therapeutic” level in the acute setting of PRES.

More than 70 cases of PRES induced by tacrolimus have been described [3, 4]. CMBs were present in only 2 observations. The first case was a 48-year-old man with past history of silicosis admitted for sudden confusion five months after a double lung transplantation [4]. The second case was a 49-year-old woman admitted 24 days after a liver transplant for generalized seizures with a blood pressure of 100/80 mmHg and a tacrolimus level of 11.6 ng/mL [5].

Although the exact pathomechanism of CMBs in our patients remains unknown, the imaging features may indicate an acute or chronic microvascular process. Tacrolimus could play a role in the occurrence of this process, regardless of the drug levels. Experimental studies conducted in vitro on mouse brain capillary endothelial cells have shown a direct cytotoxic effect of tacrolimus on endothelial cells [6]. Neuropathological study of a 40-year-old woman, deceased 2 months after a tacrolimus encephalopathy, has shown endothelial cell damage [7].

CMBs could have direct effects on cognitive function and may indicate a risk of future symptomatic intracerebral hemorrhage.

A neuropsychological and MRI monitoring including T2*-weighted sequences during the first trimester following the transplantation could be considered in patients treated with tacrolimus even without PRES symptoms.
